# Development of Dementia in Type 2 Diabetes Patients: Mechanisms of Insulin Resistance and Antidiabetic Drug Development

**DOI:** 10.3390/cells11233767

**Published:** 2022-11-25

**Authors:** Desh Deepak Singh, Ali A. Shati, Mohammad Y. Alfaifi, Serag Eldin I. Elbehairi, Ihn Han, Eun-Ha Choi, Dharmendra K. Yadav

**Affiliations:** 1 Amity Institute of Biotechnology, Amity University Rajasthan, Jaipur 303002, India; 2 Biology Department, Faculty of Science, King Khalid University, Abha 9004, Saudi Arabia; 3 Plasma Bioscience Research Center, Applied Plasma Medicine Center, Department of Electrical & Biological Physics, Kwangwoon University, Seoul 01897, Republic of Korea; 4 Department of Pharmacy, College of Pharmacy, Hambakmoeiro 191, Yeonsu-gu, Gachon University, Incheon 21924, Republic of Korea

**Keywords:** type-2 diabetes mellitus, dementia, insulin signaling, neurodegeneration, insulin resistance

## Abstract

Dementia is reported to be common in those with type 2 diabetes mellitus. Type 2 diabetes contributes to common molecular mechanisms and an underlying pathology with dementia. Brain cells becoming resistant to insulin leads to elevated blood glucose levels, impaired synaptic plasticity, microglial overactivation, mitochondrial dysfunction, neuronal apoptosis, nutrient deprivation, TAU (Tubulin-Associated Unit) phosphorylation, and cholinergic dysfunction. If insulin has neuroprotective properties, insulin resistance may interfere with those properties. Risk factors have a significant impact on the development of diseases, such as diabetes, obesity, stroke, and other conditions. Analysis of risk factors of importance for the association between diabetes and dementia is important because they may impede clinical management and early diagnosis. We discuss the pathological and physiological mechanisms behind the association between Type 2 diabetes mellitus and dementia, such as insulin resistance, insulin signaling, and sporadic forms of dementia; the relationship between insulin receptor activation and TAU phosphorylation; dementia and mRNA expression and downregulation of related receptors; neural modulation due to insulin secretion and glucose homeostasis; and neuronal apoptosis due to insulin resistance and Type 2 diabetes mellitus. Addressing these factors will offer clinical outcome-based insights into the mechanisms and connection between patients with type 2 diabetes and cognitive impairment. Furthermore, we will explore the role of brain insulin resistance and evidence for anti-diabetic drugs in the prevention of dementia risk in type 2 diabetes.

## 1. Introduction

**Figure 1:**
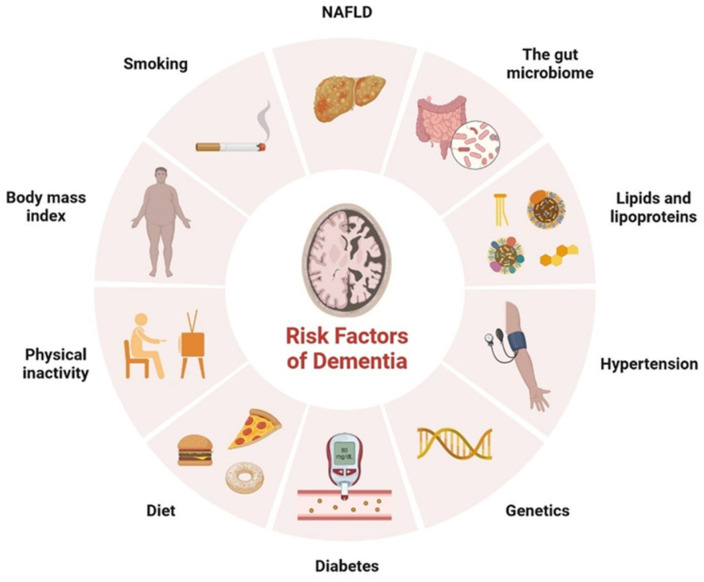
Representation of various risk factors involved in the development of dementia.

**Figure 2:**
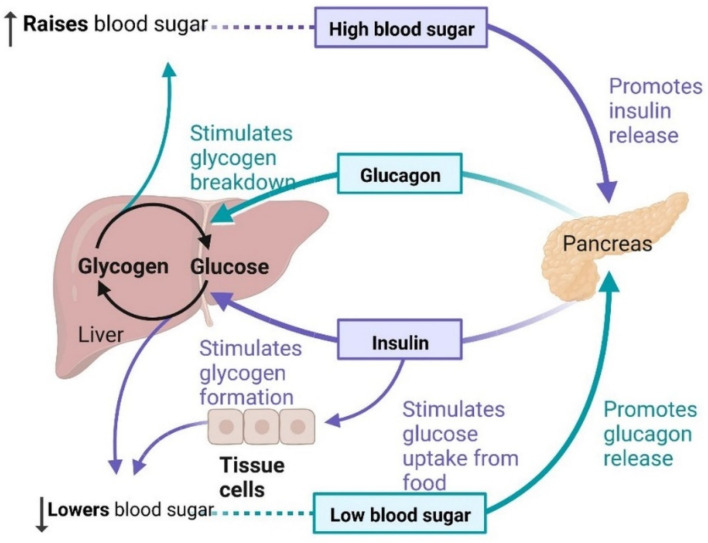
Regulation of blood glucose occurs through insulin.

**Figure 3:**
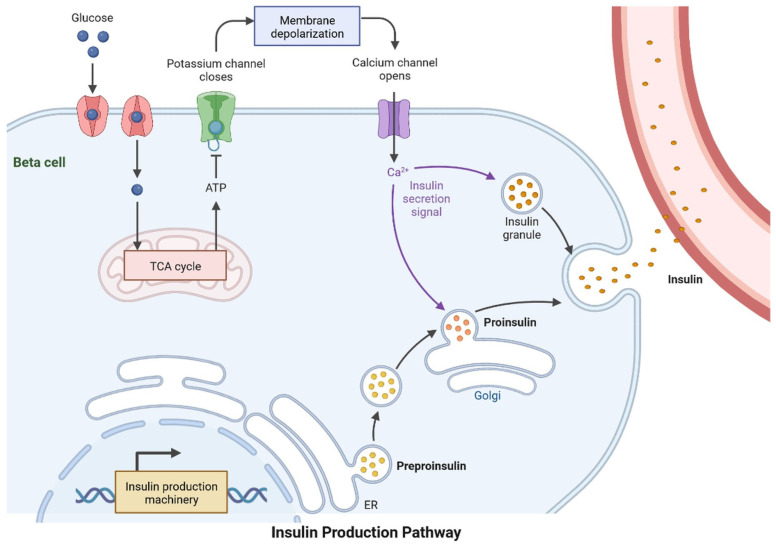
Proinsulin is transformed into insulin and C-peptide and stored in secretory vesicles, where it can be released when needed. Insulin is secreted by pancreatic beta cells to regulate glucose homeostasis. Through the GLUT transporters, glucose is freely taken in by the beta cell and metabolized to produce ATP. This triggers a series of signals inside the beta cell that are required for glucose-induced insulin production.

Type-2 diabetes mellitus (T2DM) is the most common type of metabolic disorder caused by abnormal regulation of insulin. Insulin is a non-glycosylated, 51-amino acid hormone secreted by β cells in the islets of Langerhans of the pancreas [1]. Insulin plays an important role in pathophysiological conditions and clinical complications, such as neuropathy, cardiovascular diseases, nephropathy, retinopathy, and cognitive impairment [2]. Aside from diabetes, other risk factors for dementia development include hypertension, genetics, diet, physical inactivity, smoking, and body mass index (Figure 1). Non-alcoholic fatty liver disease is involved in the development of vascular and nonvascular dementia. More than 18 million people are living with dementia globally and the number of cases is rising due to the lack of a clear mechanism between diabetes and the development of dementia. Dementia is caused by increased concentrations of the gut microbiome, higher levels of pro-inflammatory bacteria, and a reduced anti-inflammatory biome [3,4]. Hyperlipemia, which is associated with vascular disease, can develop into dementia (Figure 1) [3,4]. Blood glucose levels are regulated by negative feedback inhibition to maintain balance in the human body by pancreatic islet cells; this regulation is known as homeostasis of glucose [4,5]. Insulin lowers the blood glucose level and glucagon raises it; glucagon receptors are found in liver cells, which break down stored glycogen into glucose and release glucose in the blood, the glucose-dependent stage in human insulin regulation that does not work correctly in T2DM (Figure 2) [4,5]. Insulin is mainly synthesized by proinsulin, after which it is converted into c-peptide and insulin. C-peptide is stored in secretory granules, released on demand, and regulated by the transcription and translation processes (Figure 3) [3,4,5,6,7]. The blood–brain barrier (BBB) is crossed by insulin via a receptor-mediated mechanism [6]. During postmortem investigations, the hypothalamus has been shown to contain a significant amount of insulin [7].

The uptake of glucose in brain cells increased after activation of the ERK (extracellular signal-regulated kinases) and AKT (protein kinase B) pathways [8]. The P13K/AKT signaling pathway plays an important role in memory-encoding mechanisms and neuromodulation [9]. The insulin level in neuronal cells is determined by the rate at which it crosses the BBB by receptor-mediated transport and diffusion mechanisms. GLP-1 (glucagon-like peptide-1) regulates blood glucose levels by reducing glucagon secretion and decreasing food intake [10]. In T2DM, the GLP-1 receptor (GLP-1R) is responsible for the genes’ regulatory elements involved in neuronal survival and function. As GLP-1 agonists, they are used as targets in neurological disorders [10]. Some studies have shown that GLP-R is activated via the cAMP (cellular levels of cyclic AMP)/PKA (Protein Kinase A) pathways and is involved in neuroprotective action. The GLP-1R analog crosses the BBB and provides neuroprotection via cAMP/PKA signaling [11]. In this article, we will discuss the role of insulin signaling in the development of dementia and other neurological disorders. The discussion on GLP-1 activators and biomarkers linked to the development of T2DM and dementia revealed some remarkable points. 

## 2. T2DM and Dementia

**Figure 4:**
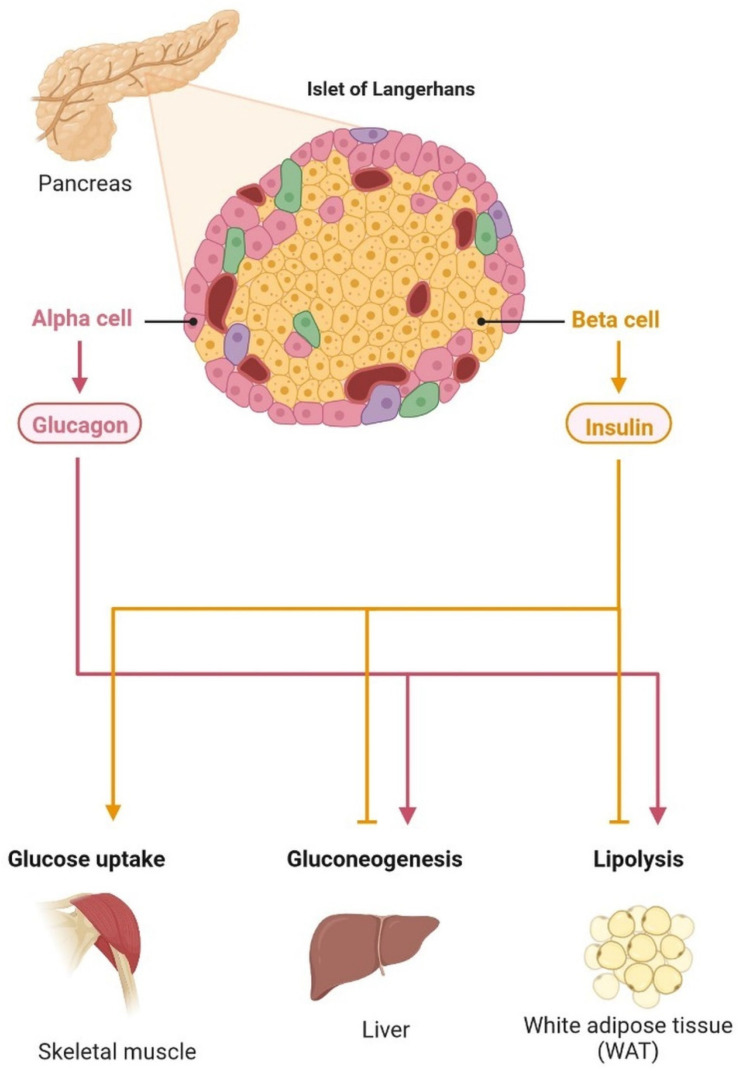
Neuronal control of peripheral insulin sensitivity and glucose metabolism. The pancreatic islets contain alpha and beta cells, which regulate glucagon and insulin, respectively. Insulin lowers the effects of glucose uptake in the skeletal muscles, liver, and brain. Blood glucose is increased by glucagon during the gluconeogenesis and lipolysis processes. The energy level is maintained by the brain in various parts of the body with a glucose homeostasis mechanism.

**Figure 5:**
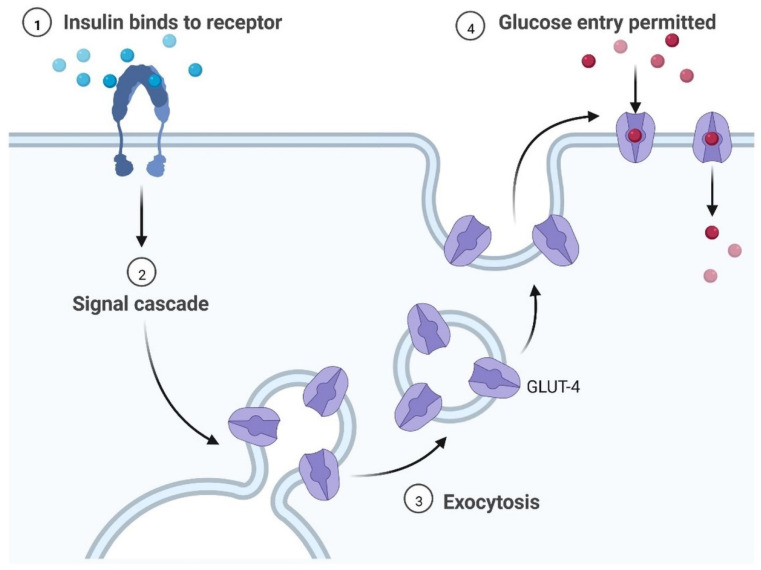
Key steps of the insulin signaling pathway, including insulin binding, signal cascade, exocytosis, and glucose entry. The regulation of signal transduction pathways depends on the signaling of extracellular chain reactions; each response is based on the course of signaling requirements. Glucose is taken up in adipose tissue and muscles via the GLUT 2 receptors in pancreatic beta cells and liver cells, with enhanced diffusion at GLUT 4 receptors. GLUT 1 and GLUT 2 allow glucose to reach cells, including the brain, retina, kidney, RBC, and other parts of the body.

T2DM patients are 1.5–2.5-times more likely to develop neurological complications than people without diabetes. Insulin resistance (IR) is linked with Alzheimer’s disease (AD), and dysregulation in the molecular mechanism of insulin secretion may lead to histopathological lesions in AD. Hyperglycemia is a major risk factor for cognitive impairment and dementia. Cognitive function may also be impacted negatively by hypoglycemia. IR is the major problem contributing to the emergence of clinical complications. The abnormalities caused by raised glucose levels were identified using the mechanisms of the insulin signaling network, including protein and lipid levels, which may cause IR. In major tissues, such as liver muscles and adipose tissues, insulin activity and its receptor regulate signaling via gene expression, phosphorylation, and vascular trafficking, increasing the consumption of nutrition, and reducing the catabolic reaction. Insulin receptors are expressed in the brain and regulate energy consumption, diet intake, behavior, and vascular function. In the last few years, molecular mechanisms and the identification and characterization of genes and proteins, circulating lipids, exosome micro-RNA, and metabolites have provided significant outcomes for IR in T2DM. AD is more common in diabetic patients than in nondiabetic patients and it is associated with a higher incidence or mortality rate [12]. The development of dementia and other neurological disorders is common in T2DM, although a sporadic form of dementia is more common (Table 1) [13]. The pancreatic islets contain alpha and beta cells, which regulate glucagon and insulin, respectively. Insulin lowers the effects of glucose uptake in the skeletal muscles, liver, and brain. Blood glucose is increased by glucagon during the gluconeogenesis and lipolysis processes. The energy level is maintained by the brain in various parts of the body with a glucose homeostasis mechanism. Neuronal control of peripheral insulin sensitivity and glucose is shown in Figure 4. Glucose intolerance has been investigated in up to 80% of dementia patients [14]. In more than 11 years of study, researchers observed a higher prevalence of dementia and AD, and a 50–100% higher risk of developing dementia in diabetic patients [15,16].The common cause of dementia is AD, an irreversible disorder, which develops slowly (Figure 4). Common signs and symptoms of dementia are loss of memory, difficulty concentrating, difficulty with familiar tasks, altered behavior, and confusion in time and place. There are various types of dementia, such as vascular dementia, Lewy body dementia, frontotemporal dementia, mixed dementia, Huntington’s disease-related dementia, and Parkinson-related dementia [17]. An epidemiological study showed that more than 55 million people are suffering from dementia and, every year, more than 10 million cases are diagnosed [18]. Dementia affects various events, such as memory, thinking, orientation, comprehension, calculation, learning capacity, language, and judgment. The brain cells affected by tau protein accumulation and plaque formation, which may cause dementia and lead to irreversible neuronal cell damage, are more common in the frontal and temporal lobes [16]. Lewy body dementia is caused by an abnormal accumulation of alpha-synuclein (-Syn) in the neurons of the substantia nigra in Parkinson’s disease [12]. Vascular dementia is caused by deformities in brain tissue, blood clots, and abnormalities of blood vessels [7]. It can be used as a component or as an interface. T2DM and dementia are associated with age and affect millions of people around the world. Patients with dementia show abnormal blood glucose levels. The regulation of signal transduction pathways depends on the signaling of extracellular chain reactions; each response is based on the course of signaling requirements. In adipose tissue and muscles, glucose enters through the GLUT 2 receptors in the beta cells of the pancreas and in the liver cells (uptake of glucose in muscle and adipose tissue via enhanced diffusion at GLUT4 receptors). GLUT 1 and GLUT allow glucose to reach cells, including the brain, retina, kidney, RBC, and other parts of the body (Figure 3, Figure 4 and Figure 5). 

### 2.1. The Glucose Transporter’s Function in Cognition 

T2DM increases the risk of cognitive impairment and IR is linked to a more rapid reduction in the memory-encoding mechanism and thinking skills [31]. Various types of genes, including SLC2A1–SLC2A14 and molecules (GLUT1–GLUT14), are actively involved in the transport of glucose in various regions of the brain and each cell type expresses multiple proteins [32]. GLUT proteins are classified into three classes. Proteins GLUT 1–GLUT 4 and GLUT 14 are classified as group I, GLUT 5, GLUT 7, GLUT 9, and GLUT 11 are classified as group 2, and GLUT 6, GLUT 8, GLUT 10, GLUT 12, and GLUT 13 are classified as group 3 [33,34]. GLUT 2 controls energy regulation, neurotransmitter release, and glucose release in glial cells. Brain stem nuclei and tanycytic, vagus motor nucleus, astrocytes, hypothalamus, arcuate nucleus, olfactory bulbs, nucleus tractus solitarius, paraventricular hypothalamic nucleus, lateral hypothalamic area, and neurons all contain GLUT 2 [34]. GLUT 3 is found in cell bodies, neurons, and dendrites; brain micro vessels; and brain astroglia cells. It has been observed that insulin accelerates the translocation of GLUT 3 and increases glucose uptake by neurons [35]. Glucose entry into the cells is carried out by GLUT 4, which is mainly found in the hippocampal region and maintains insulin regulation and improves cognitive development. It also acts as an insulin-sensitive glucose transporter [36]. There is evidence of GLUT 5 in microglial cells. The brain has a low fructose concentration, and glucose transport activity is substantially lower than that of fructose. Studies on animal models have demonstrated that fructose can pass across the BBB and be used as an energy source by brain cells [31]. The importance of GLUT 5 in the brain remains unclear and requires further investigation. GLUT 6 is involved in the nervous system’s physiological activity and transports hexoses across the membrane [34].

### 2.2. Insulin Signaling and Neuro-Complications

**Figure 6:**
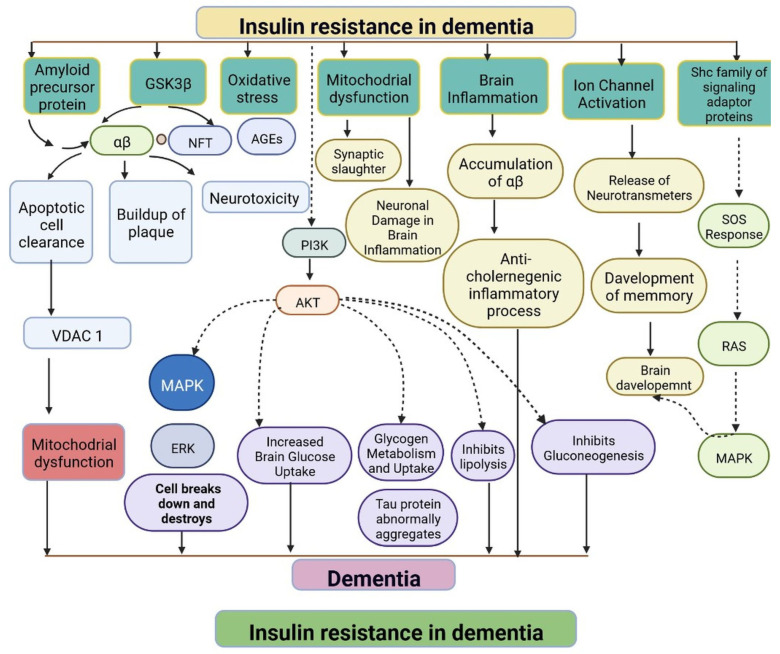
Representation of IR in dementia: resistance to insulin leads to mitochondrial dysfunction, damage to brain cells, altered brain glucose, aggregation of tau protein, inhibition of the lipolysis process, and an anticholinergic inflammatory process. The dotted lines represent inhibitory mechanisms.

**Figure 7:**
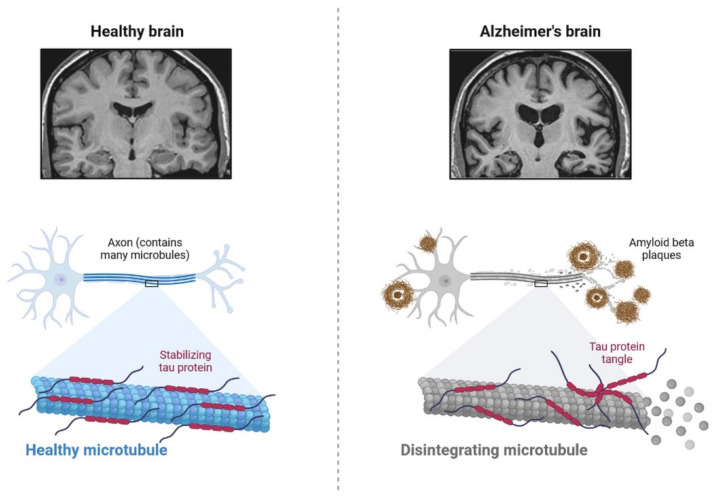
The pathological properties of the Alzheimer’s disease brain (involving tau protein) compared with that of the healthy brain, on various magnification levels. It can be adapted as a component.

**Figure 8:**
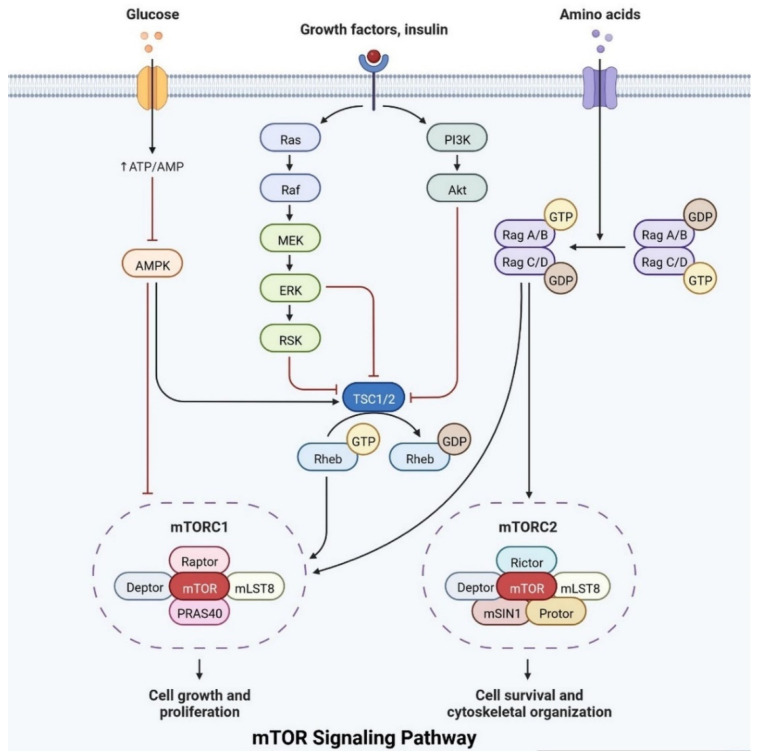
The mTOR pathway integrates growth factor signaling to regulate cellular metabolism, growth, and survival.

**Figure 9:**
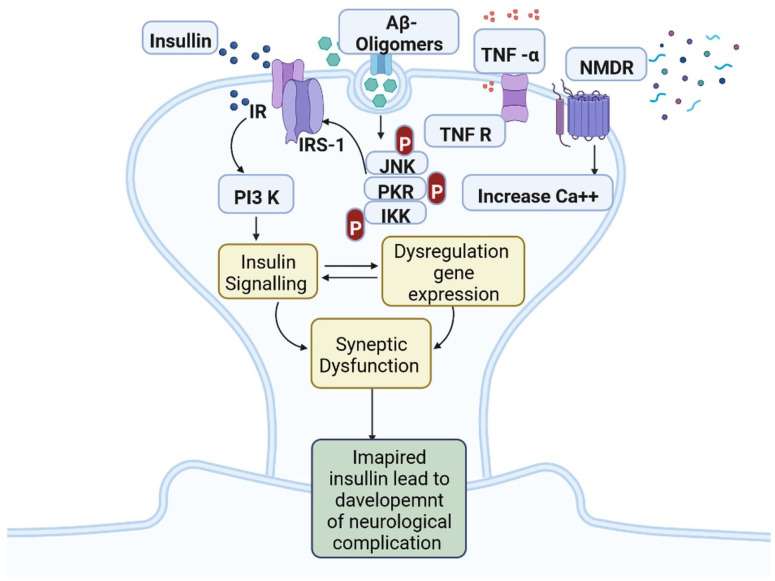
Insulin resistance and development of neuro-complications: the activation of IR autophosphorylation (IRP) leads to the tyrosine phosphorylation of IRS-1 (insulin receptor substrate 1), which activates PI3K (phosphoinositide 3-kinase) and decreases synaptic plasticity and memory.

**Figure 10:**
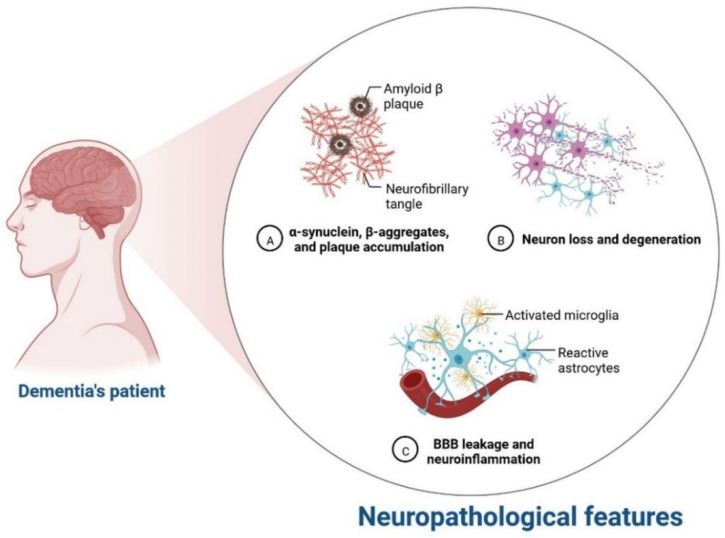
Neurological complication in dementia patients.

IR in dementia is caused by the amyloid precursor protein GSK3 (Glycogen synthase kinase 3) enzyme, involved in glycogen metabolism, oxidative stress, mitochondrial dysfunction, brain inflammation, ion channel activation, and the Shc family of signaling adaptor proteins. IR causes cell breakdown and destruction as well as increased glucose uptake, metabolism, and intake, all of which contribute to abnormal tau aggregation, inhibit lipolysis, and inhibit gluconeogenesis (Figure 6) [6]. If brain cells becoming too resistant to insulin leads to elevated blood glucose, impaired synaptic plasticity, microglial overactivation, mitochondrial dysfunction, neuronal apoptosis, nutrient deprivation, TAU phosphorylation, and cholinergic dysfunction, dementia is a group of symptoms affecting memory-encoding mechanisms, including difficulty in visual and spatial abilities, problem-solving, handling complex tasks, planning and organizing, coordination and motor functions, loss of memory, and changes in cognitive functions. Advanced dementia may develop into Alzheimer’s disease (AD). Dementia patients have synuclein aggregates and plaque accumulation, blood–brain barrier leakage, and neuroinflammation (Figure 7). Disintegrating microtubules and amyloid beta plaques are formed in AD (Figure 8). Sporadic forms of dementia are more common; both semantic and episodic memory are caused by cognitive impairment and visuospatial impairment. Motor coordination is also affected in severe cases of disease (Figure 5). [7]. IRs affect intellectual ability, increase the generation of excitability, and promote memory consolidation. IR in cognitive impairment is signified by mitochondrial dysfunction, which is involved in neurodegeneration by reducing glucose transport and inducing the formation of phosphorylated tau protein (Figure 6) [37]. The activation of IR autophosphorylation (IRP) leads to the tyrosine phosphorylation of IRS-1 (insulin receptor substrate 1), which activates PI3K (phosphoinositide 3-kinase) and decreases synaptic plasticity and memory [37,38]. The NMDARs (N-methyl-D-aspartate receptors) are activated by calcium ion channels and activated signaling is linked to the development of neurological complications. NMDAR and IR-dependent signaling molecular mechanisms resulted in amyloid oligomer accumulation, increased TNF-α release, and increased concentrations of stress-induced JNK (Jun N-terminal kinase), resulting in IRS-1 inhibitory phosphorylation. The amyloid β oligomers activate further extracellular exclusion of IRs from the cell surface. All these events block the neuronal regulation of insulin, leading to impaired synaptic plasticity (Figure 9) [37,38]. Dementia is a group of symptoms affecting memory-encoding mechanisms, including difficulty in visual and spatial abilities, problem-solving, handling complex tasks, planning, and organizing, coordination and motor functions, loss of memory, and changes in cognitive functions. Advanced dementia can progress to Alzheimer’s disease [36]. Dementia patients have synuclein aggregates and plaque accumulation, blood–brain barrier leakage, and neuroinflammation, as shown in Figure 10. Disintegrating microtubules and amyloid beta plaques are formed in AD, as shown in Figure 7.

### 2.3. Relationship between Insulin Receptor m-TOR Pathway

Insulin plays an important role in cell growth, repair, activation, dendritic development, synaptic maintenance, and neuroprotection and is actively involved in learning and memory. Various investigations have shown that changes in insulin levels in the brain lead to the development of neurological complications [39]. Insulin also activates the N-methyl-D-aspartate receptor on the cell membrane, cortical cerebral glucose metabolism, acetylcholine, and norepinephrine, which are actively involved in memory-encoding mechanisms [40]. In the cerebral cortex, IRS (insulin receptor substrate 1) is abundant in the cerebral cortex, hippocampus, hypothalamus, olfactory bulb, septum, and amygdala. Insulin signaling is also involved in regulating synaptic remodeling, which is involved in memory consolidation [9]. T2DM patients have lower brain insulin receptor sensitivity, downregulate the IRS-1, and have lower levels of insulin-like growth factors, as well as lower insulin levels in CSF [30]. Insulin signaling is controlled by mTOR pathways, as shown in Figure 8.

IRs are actively involved in memory and tau phosphorylation, due to the loss of IR receptor activity and downregulated Aβ (Amyloid beta) oligomer binding sites in the synapse [29]. The accumulation of Aβ leads to the development of AD. IGF signaling activation promotes amyloid-protein precursor (APP-A) trafficking, as well as the accumulation of amyloid processing, tau phosphorylation, and a reduction in cerebral blood flow [41]. IRs are transmembrane receptors and are made with alpha and beta subunits. Both the subunits are activated by insulin, which then activates tyrosine kinase enzymes for phosphorylation, which leads to conformational changes in their structure [38]. Changes in their structures favor binding with PI3K (phosphoinositide 3-kinases) and binding with the IRS [9]. After interaction with IRs, the inactive form of PI3K becomes active. The active PI3K enzyme is generated by PIP2 (phosphorylate phosphatidylinositol (4,5)-bisphosphate) in the cell membrane, which then causes the creation of phosphatidylinositol (3,4,5)-trisphosphate (PIP3), which activates AKT/PKB (Figure 8) [42]. Studies on animal models have revealed that IR inhibitors prevent the memory-encoding mechanism. Injecting insulin in an animal model increases the memory-encoding process [42]. IR decreases AKT activity, which inhibits GSK-3 and causes tau protein to be hyperphosphorylated [43]. AKT signaling regulates various responses at the cellular level, such as glycogen synthase kinase-3 beta (GSK3) neuronal survival and TAU phosphorylation [41]. Increasing the synthesis of GSK3β may alter the post-translational modifications in MAPs (microtubule-associated proteins), such as tau protein [41]. Dementia is also caused by mutations in APP (amyloid precursor protein). Synaptic signaling is interrupted by Aβ fibrils infiltrating into synaptic clefts [42]. Polymers of Aβ also play an important role in the development of dementia [38]. The collection of APP alters the ion channel mechanism and disrupts the altered glucose homeostasis, leading to neuronal integrity degradation and cell death [44].

### 2.4. Development of Dementia due to Genetic Modifications 

mRNA expression and the downregulation of associated receptors are linked to dementia [45]. The oxidative-phosphorylation-related genes are expressed in dementia patients. The mtDNA irregularities are associated with phenotypic variability [46]. Genetic abnormalities are caused by chromosomal defects and damage from neuronal oxidation. The development of dementia due to T2DM is linked with genetic variability [47]. APO E (apolipoprotein E) is expressed by chromosome 19 and exists in three isoforms: apo e2, apo e3, and polymorphic in nature [48]. More than 75 loci have been identified for the development of disease traits [49]. ADAM17 (A disintegrin and metalloprotease 17), ICA1 (Islet Cell Autoantigen 1), DOC2A (Double C2 Domain Alpha), DGKQ (Diacylglycerol Kinase Theta), and ICA1L (Islet Cell Autoantigen 1 Like) are the genes responsible for the regulation of APP metabolism via non-amyloid pathways. Cognitive impairment is caused by HMGB1 (High-mobility group box protein 1), RAGE (Receptor for Advanced Glycation End Products), and TLR4 (Toll-like receptor 4) in hyperglycemic conditions [50]. All these genes impair endothelial cell function and may disrupt various signaling pathways, resulting in an accumulation [51]. Dementia is also associated with the APP, PS1 (presenilin 1), and PS2 genes. Mutations in these genes cause IR in astrocytes and microglial cells [52].

### 2.5. Progression of Dementia due to Dopamine Dysregulation in Substantia Nigra

**Figure 11:**
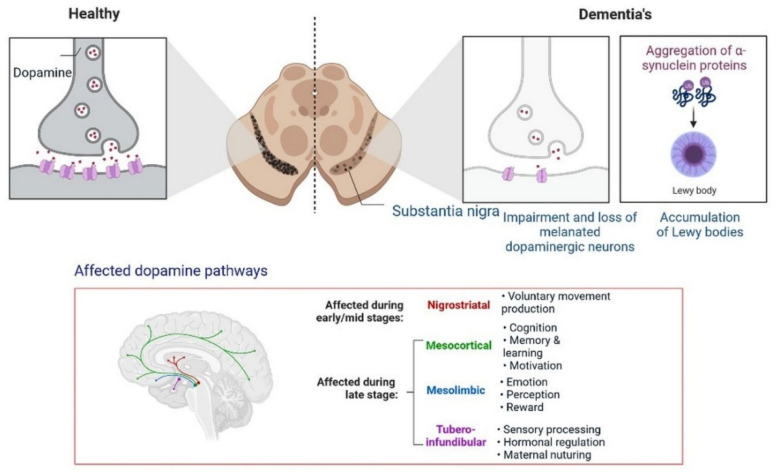
Progression of dementia due to dopamine dysregulation in substantia nigra.

Insulin secretion and glucose homeostasis serve as a basis for neural modulation. Degenerative and functional disorders of the central nervous system are directly related to dementia. Dopamine is a neurotransmitter that plays a major role in neurological complications, as shown in Figure 11.

Poor insulin activity in the brain is linked with a high level of cholinergic action, leading to the development of dementia [53].

Furthermore, the development of dementia also occurs due to alterations in the dopamine pathway in the substantia nigra. A PPAR agonist causes memory loss by increasing intracellular glucose oxidation uptake in neurons [54]. Dysregulation in cholinergic neurotransmission alters the performance of the hippocampus area, the recollection of memory, acetylcholine-induced responses, and increased ChE activity in the brain during cognitive deficits. In low amounts, choline acetyltransferase (ChAT) is observed in patients with dementia and neuronal dysfunction [55]. All these findings can be used for identification and the development of etiology and treatment options.

### 2.6. Neuronal Apoptosis in Dementia

IR and T2DM result in neuronal death. Different neurological conditions, such as Huntington’s disease, amyotrophic lateral sclerosis and dementia, Parkinson’s disease, and Alzheimer’s disease, may occur because of apoptosis [56], IGF (insulin-like growth factor) [57], increased Bax/Bcl-x ratio, hippocampal neuronal death and L caspase-3 activity, mitochondrial dysfunction, cerebral blood vessel dysfunction, myelin and axon damage, intracytoplasmic calcium deposition, and Purkinje cell damage; IGF-I, IGF-IR, and IR activity; endoplasmic dysfunction, BBB degradation; ependymal [58].

### 2.7. Significance of Ketone Bodies in Diabetes-Related Dementia

Brain cells use ketone bodies as a source of energy in situations of nutrient deprivation, after exercise, or low carbohydrates. Regulation of ketone bodies is also linked with gluconeogenesis, the tricarboxylic acid cycle, and fatty acid b-oxidation. Antioxidant responses to ketone bodies are increased [59]. Hyperglycemic conditions can reduce the activity of GABA and glutamate neurotransmitters. Cholinergic transmission was found to be dysregulated in the brain hippocampus [60]. Another neurotransmitter, dopamine, is also associated with behavior, cognition, and emotions. Reduced levels of dopamine receptors have been observed in patients with type 2 diabetes [61]. The 5-HT (5-hydroxytryptamine) neurotransmitters are associated with neuronal cell regeneration and synaptic plasticity. Glucagon-like peptide-1 hormone (GLP-1H) inhibits IR and neuroinflammation in the brain under oxidative stress. GLP1H is also involved in the regulation of synaptic plasticity and neurogenesis [62]. Ketosis increases the levels of GABA (gamma-aminobutyric acid) and excitatory glutamate and regulates the levels of serotonin and dopamine, which are linked with depression and anxiety [63]. Ketone bodies in the hydroxybutyrate form are involved in neuronal anti-apoptosis pathways and cell survival [63]. In the mouse model, fat-rich animals induce APP/PS1xdb/db deposition, whereas ketone bodies improve cognitive impairment function [60]. Ketone bodies regulate neural signaling, increase the sensitivity of insulin, reduce the effects of oxidative stress, increase synaptic activity, and maintain the level of neurotransmitter activity. Low ketone body levels may cause pathological conditions in T2DM [64]. More research is needed to understand the regulation of ketone bodies at low levels in neuroprotection and neurotoxicity at high levels in diabetes-induced dementia treatment options.

### 2.8. The Function of Mitochondria in Diabetes-Related Cognitive Impairment

**Figure 12:**
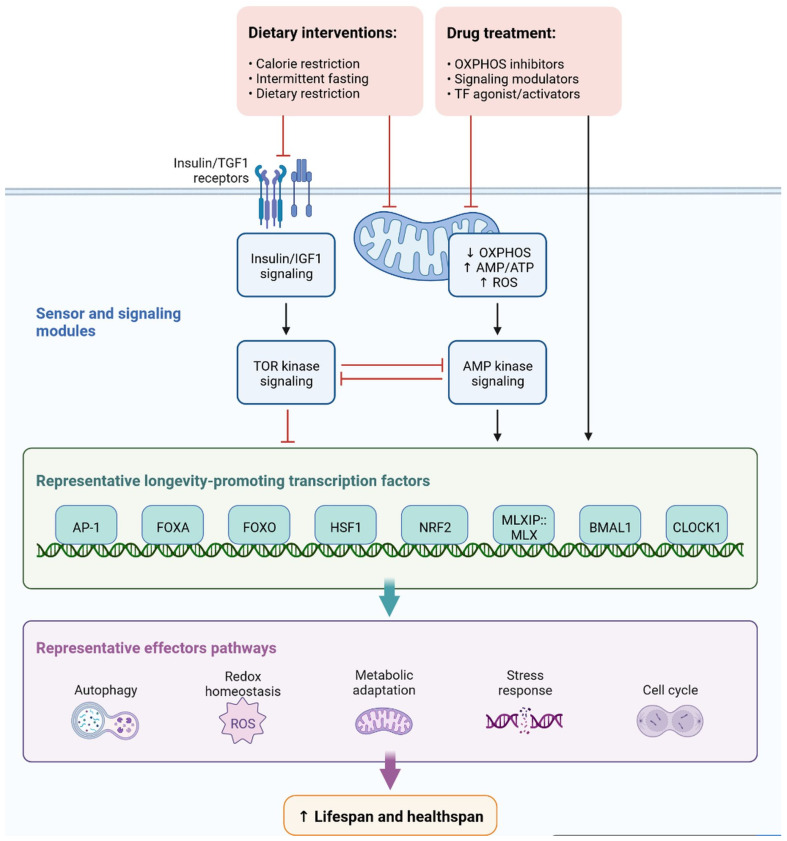
Representation of sensor and signaling molecule dietary intervention and treatment option regulated by effector pathways.

Cell signaling molecules and transcription factors play a very important role in intracellular energy metabolism in the mitochondria. Brain disorders linked with diabetes are caused by abnormalities in mitochondrial functions [65]. IR is also caused by mitochondrial dysfunction, oxidative stress, neuronal damage, decreased mETC (mitochondrial electron transport chain) activity and ATP synthesis, apoptosis, lipid peroxide accumulation, decreased glutathione peroxidase activity, ferroptosis, and An accumulation, all of which can lead to cognitive impairment [66]. Mitophagy is regulated by PINK1 (PTEN-induced kinase 1) and protects the neurons, as has been observed in animal models. PINK1-dependent mitophagy via MT2/Akt/NF-κB is achieved by melatonin, which prevents ROS (reactive oxygen species) accumulation and apoptosis (Figure 12) [67].

In diabetic mice, increased levels of LC3-II (microtubule-associated protein 1A/1B-light chain 3) and p62 (nucleoporin p62) and decreased levels of PINK1 have been observed, which leads to blocked autophagy [68]. Dephosphorylation of FUNDC1 was also observed in T2DM mice. Cognitive impairment in T2DM mice occurs due to homeostasis, impaired mitophagy, proteostasis disorder, and damage to multiple mechanisms [69]. Although various mechanisms are not completely known, we need to explore new mechanisms and pathways for the prevention and treatment of dementia. Figure 7 depicts the roles of sensor and signaling molecules, as well as transcription factors, in dietary intervention and treatment options regulated by effector pathways for early diagnosis and treatment, regulated by effector pathways for early diagnosis and treatment.

### 2.9. Progressive Dementia Due to Microglial Overactivation

**Figure 13:**
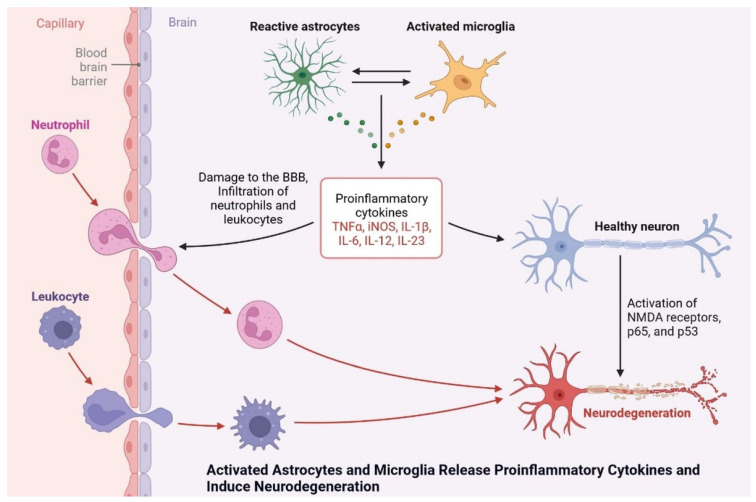
Activated astrocytes and microglia release proinflammatory cytokines and induce neurodegeneration.

Microglia cells are expressed by the AAβ protein and increase brain insulin levels due to Aβ accumulation. Protein synthesis is being studied as a potential target for drug development and dementia development. Apoptosis occurs due to the generation of ROS and AGE products [70]. Neuroinflammation is also observed due to plaque and tangle formation, oxidative stress markers, oxidized lipids and proteins, and ROS, which is insulin resistant and causes dementia. Microglial cell stimulation and increased levels of proinflammatory cytokines, such as interleukin-1, IL-6, and tumor necrosis factor, inhibit neurogenesis and cause cognitive deficits (Figure 13) [71]. More research is needed to investigate the potential of neuroinflammation treatment, and the cognitive impairment caused by diseases in their early stages.

## 3. Antidiabetic Drug Development 

Various anti-diabetic drugs are available for patients with T2DM as monotherapy or combination therapy [72]. Most of the drugs mainly target neurological, cognitive, and cardiovascular clinical complications. Antidiabetic drugs are classified based on their mechanism of action [73]. The clinical evaluation of anti-diabetic drugs in diabetic patients with cognitive dysfunction was investigated in various studies (Table 2).

### 3.1. Glucose–Sodium Co-transporter 2 

SGLT2 (sodium–glucose co-transporter 2) is an inhibitor of the reabsorption of glucose in the renal tubules. In a mouse model, the efficacy of empagliflozin and dapagliflozin was investigated. In treated mice, plaque burden and neuronal deactivation have been observed [73]. In another study, improved mitochondrial function and cognitive impairment were observed in rats treated with dapagliflozin [74].

### 3.2. Pioglitazone 

Pioglitazone has been shown to improve cognitive impairment and reduce tau protein deposition in triple-transgenic mice [75]. Pioglitazone has also been shown to activate microglia in another model [76]. Pioglitazone treatment also normalizes metabolic and vascular functions, reduces IR, and maintains ROS (reactive oxygen species) in the hippocampus and cerebral cortex for learning and memory [77].

### 3.3. Rosiglitazone 

In various investigations, the clinical efficacy of rosiglitazone was observed. A randomized double-blind study showed improved cognitive impairment in early-stage AD patients without the ApoE4 allele [78]. Treatment with rosiglitazone has shown mitochondrial biogenesis in the mouse brain [79] and combination therapy with metformin has shown stable results [80].

### 3.4. Metformin

Metformin is an oral hypoglycemia drug; various clinical investigations are carried out on cognitive dysfunction in T2DM. Metformin in combination with sulfonylureas has reduced dementia by up to 35% [81]. Long-term metformin treatment has been linked to the development of Alzheimer’s disease. No dementia or neurological complications have been observed with other drugs, such as thiazolidinediones, sulfonylureas, or insulin.

### 3.5. Thiazolidinediones 

Thiazolidinediones are PPAR agonists that increase insulin secretion in T2DM patients. Two drugs, namely rosiglitazone and pioglitazone, belong to the thiazolidinediones. Clinical efficacy has been shown to improve cognitive impairment [82].

### 3.6. GLP-1-Based Therapies 

It has been observed that the expression of GLP-1 in mice improves cognitive function. Glp-1-expressing mice also had severely impaired LTP in the CA1 area of the hippocampus. GLP-1 plays an important role in memory formation [83].

### 3.7. GLP-1 Analogs

Exendin-4 reduced HbA1c and cerebrovascular A in a 16-week trial in 5XFAD mice (3xTgAD). Exendin-4 also reduces neuronal apoptosis, activates the CREB transcription factor, and induces BDNF activity. Another 16-week study in 5XFAD mice looked at maintaining mitochondrial functioning and cognitive impairment [84]. 

### 3.8. Liraglutide 

Improved cognitive impairment has been observed after a 3-month administration of liraglutide in a mouse model. Another study analyzed a 28-day liraglutide treatment in 3xTg-AD female mice and observed that it prevented memory impairment [85]. In treated liraglutide HFD-fed mice or ob/ob mice, neurogenesis and hippocampal synaptic plasticity were significantly improved [86].

### 3.9. Dulaglutide and Lisisenatide 

The clinical efficacy of dulaglutide was investigated in 371 sites in 24 countries and reduced cognitive impairment in T2DM by 14%. Lixisenatide was found to be effective against plaque deposition and neuroinflammation in the brains of tau mice after 2 months of treatment. In HFD-fed mice, lixisenatide has been shown to reduce IR and neurogenesis [87].

### 3.10. New GLP-1 Analogs 

GLP-1 and gastric inhibitory peptide (GIP) receptor agonists may be more effective in the treatment of Alzheimer’s disease [88]. In an intracerebroventricular STZ-induced IR AD rat model, working memory and spatial memory deficits were reversed by DA5-CH, a new dual GLP-1/GIP receptor agonist [89]. In the 3xTg mouse model, a new GLP-1/GIP, and GIP were found to enhance working memory.

### 3.11. DPP-4 Inhibitors 

Current diabetic treatments, such as sitagliptin, vildagliptin, alogliptin, and linagliptin, all use different forms of DPP-4 inhibitors to maintain the levels of endogenous GLP-1. DPP-4 inhibitors have been shown to greatly prevent dementia with metformin and thiazolidinedione [90].

### 3.12. Linagliptin A

In a 3xTg-AD mouse model, linagliptin treatment for 8 weeks resulted in a notable improvement in cognitive impairment. Linagliptin also lessens the development of amyloid beta-amyloid and neuroinflammation [91]. Furthermore, linagliptin also increases cerebral blood flow and cognitive decline in a tauopathy mouse model. The DPP-4 inhibitor also increased the levels of the tight-junction protein claudin-5 and prevented neuronal loss in the hippocampus and cortex of these mice. 

### 3.13. Alogliptin 

Alogliptin mainly activates CREB in insulin-positive β cells of the islets and increases anti-apoptotic bcl-2. Another study carried out in Zucker diabetic rats for 10 weeks showed neuroprotective proteins in the brain and reduced the level of inflammatory markers [92].

### 3.14. Vildagliptin 

A 6-month clinical trial with vildagliptin improved HbA1c and cognitive impairment in T2DM patients [93]. In HFD rats, combined therapy with pioglitazone improved dendritic spines in the CA1 hippocampus and reduced apoptosis of hippocampal neurons [93].

### 3.15. Sitagliptin 

The efficacy of sitagliptin has been studied in animal models. Improved cognitive impairment and reduced plaque deposition have been observed in APP/PS1 mice after oral administration of sitagliptin for 8 weeks [94]. Furthermore, they reduced the activity of inflammatory markers and suppressed white-matter lesions.

## 4. Conclusions

Determining the mechanisms associated with T2DM can lead to new approaches to dementia’s early detection and treatment. The link between T2DM and cognitive dysfunction is mainly reliable and a study on humans and animals can explore the most effective mechanism that could be useful. There is a very tough puzzle to solve for T2DM and the degeneration of neurons. Neurofibrillary tangles and neurotic plaques, cerebrovascular abnormalities, decreased brain volume, markers of white-matter injury, retinal measures, retinal nerve fiber, retinal vascular tortuosity, and fluid markers for gliosis and neurodegeneration are all targets for cognitive dysfunction. The development of glycoproteomic-based tools may be useful for the identification of novel biomarkers for early diagnosis and treatment. The development of novel treatment options for T2DM in dementia IR is developed due to altered insulin signaling, which is essential for energy metabolism, neuronal growth, neuroprotection, and synaptic plasticity. Peripheral and central IR are common in neurological disorders and can be used as potential targets for intervention in and treatment of dementia. Various clinical trials have improved cognition. As a result, insulin treatment must be tested in a variety of contexts, including known resistance or patients with T2DM, screening, and including patients at all stages. We can reduce the global burden of dementia by applying these promising areas of research and novel approaches for patients with T2DM and dementia. 

## Figures and Tables

**Table 1:** The common occurrence of neurological complications in insulin-resistant patients.

S.N.	Disease	Type of Study	Disease Model	Concerned Area of the Brain	Factor Involvement	Outcome of the Study	References
1.	Type-2DM	Clinical study Prospective population-based cohort study	T2DM patients	Cerebrum	Risk of dementia (hazard ratio-HR, 1.3 to 2.8), ↑ (Increased) risk of Alzheimer’s disease (HR 1.2 to 3.1).	Increased risk of dementia	[19]
2.	T2DM	Clinical study Longitudinal cohort study	16,667 patients, Mean age: 65 years Morbidity: T2DM, without prior diagnoses of dementia	Cerebrum	VCI (Vascular cognitive impairment) Incident VCI (RR-Relative risk): 1.62; 95% CI-Confidence interval: 1.12–2.33)	Increase extracellular plaques deposition	[20]
3	Abnormal insulin Level in AD	Clinical study	Patients with, abnormal insulin Level in Alzheimer’s Disease (AD) and healthy adults	Blood plasma, Cerebrospinal fluid	↓ (Decrease) CSF (cerebrospinal fluid) insulin concentration led to more advanced dementia. ↑ Plasma insulin levels signified more advanced dementia.	Increased risk for the development of AD	[21]
4.	Type-2DM	Longitudinal study	2322 adult male participants, Mean age: 50 years old	Hippocampus, cerebellum	↑risk of dementia, and VD with severe systolic hypertension or heart disease.	Increased risk of AD dementia	[22]
5	Type-2DM	Epidemiological study	Cohort study 1892 Jewish male civil servant, Mean age: 82 years	Cerebrum	↑dementia risk factor;(HR = 2.83, 95% CI = 1.40 to 5.71).	Increase the pathogenesis of dementia	[23]
6	Type-2DM	Population-based Longitudinal cohort study	2322 adult male participants, Mean age: 50 years old	Hippocampus is the affected part.	Increased risk of AD dementia (HR = 1.31; 95% CI, 1.10–1.56), VD (HR = 1.45; 95% CI, 1.05–2.00).	Increase the risk of cognitive impairment.	[24]
7	Type-2DM	Clinical study Retrospective longitudinal cohort study	Adult diabetic patients with prior hypoglycaemia had a significantly higher rate of dementia.	Cerebral cortex	One episode (HR = 1.26; 95% CI = 1.03–1.54)	Increased risk of hypoglycaemia	[25]
8	Type-2DM	Clinical study Retrospective longitudinal cohort study	Age >65 years, diagnosed with T2DM, with no prior diagnosis of dementia	Cerebral cortex	One episode (HR = 1.26; 95% CI = 1.03–1.54) was associated with an increased risk.	Hypoglycaemia is associated with a higher risk of dementia.	[26]
9	Type-2DM	longitudinal studies	6184 subjects with diabetes and 38,530 subjects without diabetes	Cerebral cortex and hippocampus	Increased risk of any dementia (RR: 1.51, 95% CI: 1.31–1.74) and increase risk of MCI (RR: 1.21, 95% CI: 1.02–1.45).	Diabetes is a risk factor for incident dementia (including AD, VD, and any dementia).	[27]
10	Type-2DM	Prospective study	1066 men and women with T2DM Age: 60–75 years	Cerebral and hippocampus	Severe hypoglycaemia is associated with impaired initial cognitive ability and cognitive decline.	Diabetes is a risk factor for incident dementia	[28]
11	Type-2DM	Retrospective national record linkage cohort study	343,062 people with T1DM; 1,855,141 people with T2DM	Cerebral and hippocampus	Risk for developing dementia in T1DM people (RR = 1.65; 95% CI 1.61, 1.68).	Risk of developing dementia in the hippocampus.	[29]
12	Type-2DM	Long-term prospective cohort study	135 autopsies of residents of Hisayama town (74 men and 61 women)	Cerebral and hippocampus	The risk of neurotic plaque formation	Increased risk of AD pathology.	[30]

**Table 2:** Antidiabetic drug list, including mechanisms of action, significance, and risk factors.

Type of Class	Antidiabetic Agent	Types of Patients	Mechanism of Action	Major Role	Risk	Contraindications
Potent inhibitor of the sodium-glucose cotransporter 2 (SGLT-2)	Canagliflozin	Patients with T 2 DM without renal failure	Polyuria and glycosuria are caused by glucose reabsorption in the glomerulus tubule of the kidney and reversible inhibition of SGLT-2 in the proximal tubule of the kidney.	Inhibition of SGLT-2 in the kidney	Glucosuria Ketoacidosis and excessive urination Weight loss	Chronic kidney disease
	Dapagliflozin	Used in patients to improve glycaemic control with T2DM				
	Empagliflozin	Used to reduce the risk of cardiovascular death in patients with T2DM				
Sulfonylureas	First generation Chlorpropamide	promote insulin release with T2DM	Prevent calcium influx, insulin secretion, transmembrane depolarization, and ATP-sensitive potassium channels in pancreatic cells.	Increase insulin secretion from pancreatic β cells	hypoglycemia Disulfiram-like reaction agranulocytosis, hemolysis	Cardiovascular comorbidity Obesity Severe renal or liver failure
	First generation Tolbutamide	promote insulin release with T2DM				
	Second generation Glyburide	Used in elderly patients with diabetes				
Dipeptidyl peptidase-4 (DPP-4) inhibitors	Saxagliptin	Patients with Renal Impairment with T2DM	Indirectly increase the effect of endogenous renin-angiotensin by inhibiting the DPP-4 enzyme, which disintegrates GLP-1, insulin secretion, glucagon secretion, and delayed stomach emptying.	Inhibit GLP-1 degradation	Pancreatitis Nasopharyngitis, Headache, dizziness Arthralgia Edema	Liver failure Renal failure
	Sitagliptin	Used to reduce blood sugar levels in adults with T2DM				
	Linagliptin	Used to reduce blood sugar levels in adults with T2DM				
Meglitinides	Nateglinide	Increase the secretion of insulin released by the pancreas with T2DM	ATP-sensitive potassium channels are blocked, cell membranes are depolarized, calcium influx occurs, and insulin secretion is increased.	Increase insulin secretion from pancreatic β cells	hypoglycemia Weight gain	Severe liver failure
	Repaglinide	It is used when Insulin is not synthesized by the body in a patient with T2DM				
Thiazolidined iones	Pioglitazone	Used to reduce the risk of cardiovascular death in patients with T2DM	Enhancing adipokine transcription, peroxisome proliferator-activated receptors (PPARs) activation can decrease IR. increased insulin secretion.	Reduce IR	Edema Cardiac failure Weight gain Osteoporosis	Congestive heart failure Liver failure
	Rosiglitazone	Increase the secretion of insulin released by the pancreas with T2DM				
Amylin analogs	Pramlintide	used to reduce blood sugar levels in adults with T2DM	The secretion of insulin is stimulated by decreased glucagon release, a slower pace of stomach emptying, and an elevated level of satisfaction.	Decrease glucagon release	hypoglycemia Nausea	Gastroparesis
Metformin	Biguanides	preventing the production of glucose in the liver and improving insulin sensitivity in T2DM patients	Metformin increases AMPK activity, lowers cAMP, reduces the production of gluconeogenic enzymes, improves insulin sensitivity (via effects on fat metabolism),	Enhances the effect of insulin	Lactic acidosis Weight loss Gastrointestinal Diarrhoea,	Before the administration of iodinated contrast medium and major surgery, metformin must be stopped.
Glucagon-like peptide-1 (GLP-1) agonists (incretin mimetic drugs	Exenatide	used to reduce blood sugar levels in adults with T2DM	Increased food intake stimulates the digestive tract’s endocrine cells to generate GLP-1, which the enzyme DPP-4 breaks down to boost insulin secretion.	Stimulate the GLP-1 receptor	pancreatitis cancer Nausea	Gastrointestinal motility disorders
	Liraglutide	used to reduce T2DM by improving insulin sensitivity				
	Albiglutide	used to reduce blood sugar levels in adults with T2DM				
